# Extracellular S100A4 as a key player in fibrotic diseases

**DOI:** 10.1111/jcmm.15259

**Published:** 2020-04-19

**Authors:** Zhenzhen Li, Yanan Li, Shuangqing Liu, Zhihai Qin

**Affiliations:** ^1^ Medical Research Center The First Affiliated Hospital of Zhengzhou University Zhengzhou University Zhengzhou China; ^2^ School of Medicine Ruijin Hospital Shanghai Jiaotong University Shanghai China; ^3^ Key Laboratory of Protein and Peptide Pharmaceuticals CAS‐University of Tokyo Joint Laboratory of Structural Virology and Immunology Institute of Biophysics Chinese Academy of Sciences University of the Chinese Academy of Sciences Beijing China

**Keywords:** biomarker, damage‐associated molecular pattern, extracellular S100A4, Fibrosis, inflammation

## Abstract

Fibrosis is characterized by fibroblast activation, extracellular matrix (ECM) accumulation and infiltration of inflammatory cells that sometimes leads to irreversible organ dysfunction. Considerable evidence now indicates that inflammation plays a critical role in the initiation and progression of organ fibrosis. S100A4 protein, a ubiquitous member of the S100 family, has recently been discovered as a potential factor implicated in fibrotic diseases. S100A4 protein is released at inflammatory site and has a certain biological function to promote cell motility, invasion, ECM remodelling, autophagy and angiogenesis. In addition, extracellular S100A4 is also a potential causation of inflammatory processes and induces the release of cytokines and growth factors under different pathological conditions. Elevated S100A4 level in patients’ serum closely correlates with disease activity in several fibrotic diseases and serves as a useful biomarker for diagnosis and monitoring disease progression. Analyses of knockout mouse models have identified a functional role of extracellular S100A4 protein in fibrotic diseases, suggesting that suppressing its expression, release or function might be a promising therapeutic strategy. This review will focus on the role of extracellular S100A4 as a key regulator of pro‐inflammatory signalling pathways and its relative biological processes involved in the pathogenesis of fibrosis.

## 1 INTRODUCTION

An appropriate wound repair response is the premise of restoring the homeostasis of the damaged tissue. Wound maladaptation caused by chronic inflammation can result in fibrosis.^1^, ^2^ Fibrosis is the formation of excess fibrous connective tissue in an organ or tissue in a reparative or reactive process. Fibrosis is characterized by fibroblast activation, extracellular matrix (ECM) accumulation and infiltration of inflammatory cells that sometimes leads to irreversible organ dysfunction.^3^ Considerable evidence now indicates that inflammation plays a critical role in the initiation and progression of organ fibrosis.^4^ Inflammation is an important part of the body's natural defence system in which immune cells participate. Inflammation can resist the damage caused by pathogens, various drugs and stress. However, persistent inflammation is associated with a variety of different pathological conditions including organ fibrosis.^5^, ^6^, ^7^

S100A4 (also called fibroblast‐specific protein 1 (Fsp1)) is a member of the S100 calcium‐binding protein family. The most well‐known function of S100A4 is to induce and promote tumour metastasis.^8^ Apart from this function, S100A4 was also involved in the pathophysiology of fibrotic, inflammatory and autoimmune disorders.^9^, ^10^ Like other members of the S100 family, S100A4 functions both intra‐ and extracellularly. Within the cell, the presence of S100A4 is related to apoptosis, migration and maintenance of cell stemness.^11^, ^12^ Extracellular S100A4 can activate different processes mainly through inducing the expression and secretion of pro‐inflammatory cytokines, growth factors and matrix metalloproteinases (MMPs), as well as stimulating pro‐inflammatory related pathways.^8^ Therefore, the extracellular function of S100A4 is mainly due to its pro‐inflammatory and pro‐metastatic activities.

**Figure 1:**
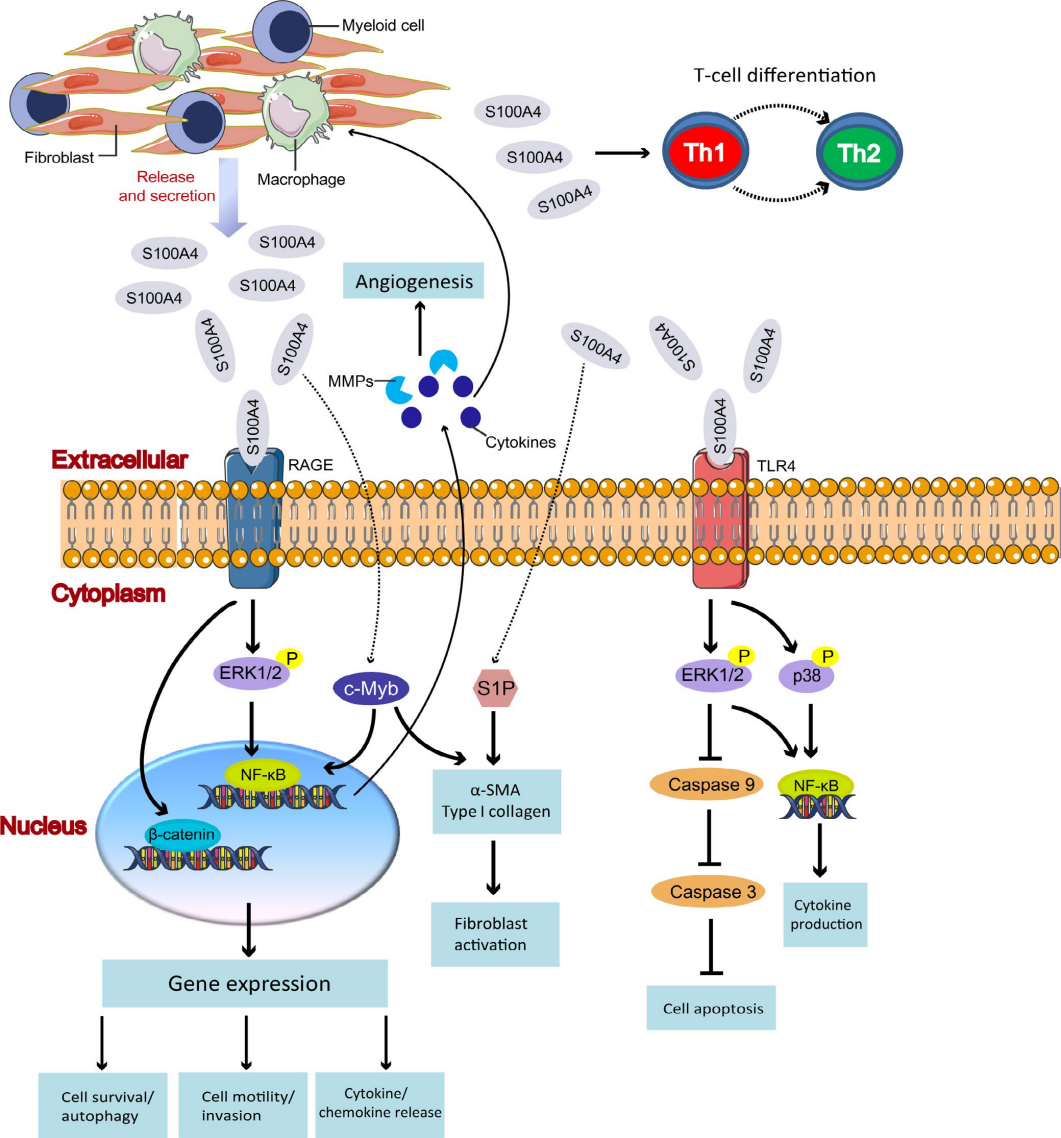
Functions of extracellular S100A4 protein. S100A4 can be released into the extracellular space by fibroblasts, macrophages, lymphocytes and myeloid cells. The expression of extracellular S100A4 leads to increased phosphorylation of ERK1/2 and activation of NF‐κB through the RAGE‐dependent regulation, which is associated with cell motility, invasion, cell survival and inflammation. The consequent sustained release of pro‐inflammatory cytokines and MMPs promote angiogenesis. Besides, extracellular S100A4 interacted with RAGE exerts an inhibitory effect on autophagy through activating β‐catenin signalling pathway. On the other hand, extracellular S100A4 activates TLR4/ERK1/2 pathway to abrogate caspase‐9‐dependent apoptosis. Meanwhile, extracellular S100A4 induces inflammatory response partly mediated by TLR4 and through the activation of NF‐κB axis, the kinases p38 and ERK1/2. In addition, extracellular S100A4 increases the expression of α‐SMA through activating of c‐Myb and S1P pathway. Extracellular S100A4 also can affect T cell differentiation by the alteration of T cell polarization balance toward the Th2 phenotype. RAGE, receptor for advanced glycosylation end products; TLR4, Toll‐like receptor 4; MMPs, matrix metalloproteinases; ERK1/2, extracellular signal‐regulated kinase; NF‐κB, nuclear factor kappa‐light‐chain‐enhancer of activated B cells；S1P, sphingosine‐1‐phosphate; α‐SMA, α‐smooth muscle actin

Here, we summarize the role of extracellular S100A4 protein enhancing inflammation in the pathophysiology of fibrotic diseases (Figure 1) and discuss how extracellular S100A4 protein might be used or targeted in future strategies to diagnose and treat these diseases.

## 2 ROLE OF EXTRACELLULAR S100A4 IN FIBROTIC DISEASES

It is widely accepted that there is a close relationship between S100A4 and non‐tumour pathophysiology, especially organ fibrosis. Up to now, S100A4 has been implicated in the development of many organ fibrosis, such as kidney fibrosis, liver fibrosis, pulmonary fibrosis and artery diseases, cardiac hypertrophy and fibrosis and rheumatoid arthritis.^10^, ^13^ Here, we will review the recent findings on role of extracellular S100A4 in the pathogenesis of various fibrotic diseases.

In a model of liver fibrosis, Christoph H. Österreicher et al revealed S100A4 expression by macrophages.^14^ Here, S100A4‐positive cells did not express the classical fibroblast markers, like α‐smooth muscle actin (α‐SMA) or desmin, but F4/80 and other markers of monocytic lineage as evaluated by genetic lineage tracing and immunofluorescence staining. Our previous study found that extracellular S100A4 accumulated in the liver during fibrosis progression.^15^ Among the S100A4‐GFP‐positive cells, 97.9 ± 0.76% were CD45‐positive, 86.0 ± 2.34% were CD11b‐positive and 79.5 ± 1.54% were Ly6C^hi^. Wright's staining of sorted GFP‐positive cells confirmed that these cells possess the characteristic morphology of monocytes/macrophages. These results indicated that S100A4‐positive cells are a subpopulation of macrophages in the liver. In vitro, exogenous S100A4 induces the up‐regulation of α‐SMA in hepatic stellate cells through activating of the c‐Myb signal pathway. Selective depletion of S100A4‐positive cells or knockdown of S100A4 by RNA interference in vivo strongly reduces collagen deposition in the injured liver. Apart from the liver, we also found S100A4^+^ monocytes/macrophages in the bone marrow, peripheral blood and in spleen tissues. Whether CD45^+^/S100A4^+^ positive macrophages in fibrotic areas of the liver originate from bone marrow‐derived fibrocytes needs further exploration.

In pulmonary fibrosis, fibroblasts are largely responsible for the production and deposition of interstitial collagen and other ECM molecules.^16^ It is not well defined where the activated fibroblasts come from during lung fibrosis. The resident fibroblasts from interstitium, progenitor cells from bone marrow and transdifferentiation of epithelial cells to myofibroblasts were considered to be potential sources of activated fibroblasts.^17^ The crucial role of intracellular S100A4 for fibroblasts especially in human pulmonary fibrosis renders intracellular S100A4 a good marker for this cell type in investigating the pathogenesis of pulmonary fibrosis.^18^, ^19^ Interestingly, our recently published data found increased S100A4 protein level in human bronchoalveolar lavage fluid of idiopathic pulmonary fibrosis (IPF) patients.^20^ Our data also showed that during the progress of bleomycin‐induced pulmonary fibrosis, a population of S100A4^+^CD11b^+^F4/80^+^ macrophages accumulated in lung tissue and correlated very well with the development of lung fibrosis. This special subpopulation of macrophages was observed to secret S100A4 into extracellular space. In vitro studies revealed that extracellular S100A4 could activate both mouse and human lung fibroblasts by up‐regulation of α‐SMA and type I collagen, during which sphingosine‐1‐phosphate (S1P) increased. Our research suggests that extracellular S100A4 or S100A4‐positive macrophages within the lung as promising targets for early clinical diagnosis or therapy of IPF. Coincidentally, another research from Zhang et al also supports our view that S100A4 is produced and secreted by M2 polarized alveolar macrophages and enhances the proliferation and activation of lung fibroblasts.^21^ These two studies have different understandings about the source of extracellular S100A4. Whether S100A4^+^CD11b^+^F4/80^+^ macrophages and M2 macrophages belong to the same group of cells remains to be confirmed by further research.

Rheumatoid arthritis (RA) is characterized by inflammatory infiltration, activated fibroblasts and tissue remodelling.^9^ In this regard, arthritis may share underlying mechanisms with fibrosis. Proliferating synovial fibroblasts from rheumatoid arthritis patients express high levels of S100A4.^22^ Fibroblast, immune and vascular cells result in the production of S100A4 in synovial tissue consistent with the high concentration of S100A4 protein in RA patients’ plasma and synovial fluid. Serum S100A4 levels are increased with the aggravation of radiographic damage and disease progression. High serum S100A4 levels are also linked to a poor clinical response to anti‐tumour necrosis factor α (TNF‐α) therapy.^23^ Additionally, persistently high serum S100A4 levels predict poor treatment outcomes and S100A4 may thus represent a promising biomarker for assessing treatment response in patients with RA.^24^ Accelerated release of MMP‐13 from chondrocytes cell line or cartilage explants upon treatment with recombinant exogenous S100A4 protein confirmed the link between S100A4 and pathologic tissue remodelling in this disease.^25^

The variety of diseases and S100A4‐producing cell types leave many open questions, but clearly place extracellular S100A4 as a major contributor to the progression of fibrosis in chronic inflammatory diseases and make it be a candidate therapeutic target for antifibrotic treatment.

## 3 CHARACTERISTICS OF EXTRACELLULAR S100A4

### 3.1 S100A4 is secreted by uncertain mechanisms

Like other members of the S100 family, the S100A4 protein exists in both intracellular and extracellular. The intracellular S100A4 protein is mainly localized in the cytoplasm and nucleus. Changes in intracellular S100A4 function depend on the morphology of the protein and are pre‐conditioned through the interaction and regulation of multiple intracellular protein targets located in different cell compartments. As the role of intracellular S100A4 has been widely described, we solely focus on extracellular S100A4 and related mechanism (for recent reviews on intracellular S100A4, please see ^8^, ^10^, ^13^). With the in‐depth understanding of S100A4 protein, more and more studies have found that extracellular S100A4 serves as a more essential role. Increasing evidence showed that S100A4 in extracellular space is closely related to the progression of tumour metastasis ^26^, ^27^ and other human inflammatory diseases.^28^, ^29^, ^30^ In fact, under physiological conditions, S100A4 is rarely expressed in certain cell types, such as lymphocytes, monocytes, neutrophils, stem cells and fibroblasts.^28^, ^31^, ^32^, ^33^ Importantly, only a very small amount of extracellular S100A4 protein can be detected in body fluids under physiological conditions.^28^ Functionally, it has been found that extracellular S100A4 can induce the release of pro‐inflammatory factors, such as cytokines, serum amyloid (SAA), and MMPs. It stimulates neuron differentiation, survival and neurite outgrowth, and promotes cell invasion, angiogenesis and chemotaxis.^8^, ^34^

Although there is a lot of evidence about the function of extracellular S100A4 in vivo and in vitro, there are few reports on the mechanism of S100A4 secretion.^28^, ^35^ The S100A4 protein cannot be secreted through the classical endoplasmic reticulum (ER)‐Golgi‐dependent pathway due to the lack of a secretion signalling peptide.^36^, ^37^ Lysosomes, which are involved in the secretory process of different cell types, have also been excluded from alternative mechanisms. Surprisingly, studies have found that cytokine‐mediated pathways play an important role in stimulating S100A4 secretion in different types of normal cells and tumour cells. For example, the cytokine interleukin (IL)‐7 can regulate the secretion of S100A4 in chondrocytes, but which is irrelative for the classic Golgi pathway.^36^ Another cytokine, regulated on activation in normal T Cell expressed and secreted (RANTES) can stimulate the secretion of extracellular S100A4, which is associated with microvesicles (MVs) that shed from plasma membranes of tumours and normal cells.^37^ The importance of the RANTES‐S100A4 axis has been further verified by planting RANTES‐overproducing tumour cells in wild‐type and S100A4 gene‐deficient mice. Results showed that tumour cell‐derived RANTES had a significant effect on the increased level of extracellular S100A4 in blood circulation.^37^ In addition, researchers observed the significant increase of S100A4 in the condition medium of Burkit lymphoma B cells (Namalwa cells) transformed with transcription factor Octamer transcription factor 1 (Oct‐1A), especially its isoform Oct‐1X.^38^ However, these rather sparse shreds of evidence seem not sufficient to explain the omnipresence of exogenous S100A4. Other cell type‐specific mechanisms for S100A4 release are possible.

### 3.2 Possible underlying mechanisms of extracellular S100A4 in fibrosis pathophysiologies

Cell movement and invasion play an important role in tumour metastasis and inflammation, which are processes closely related to epithelial‐mesenchymal transition (EMT).^39^ As the most widely studied putative receptor, the interaction of receptor for advanced glycation end‐products (RAGE) and S100A4 has an obvious effect on cell movement and are capable of modulating the motility of thyroid cancer cells, colon adenocarcinoma cells and vascular smooth muscle cells by regulating mitogen‐activated protein kinase/extracellular signal‐regulated kinase (MAPK/ERK) pathway and hypoxia signalling.^40^, ^41^, ^42^ In addition, recombinant S100A4 protein is confirmed to stimulate endothelial cell motility and invasion ability by promoting MMP‐13 mRNA and protein expression.^43^

ECM is a highly dynamic structure that is present in all tissues and undergoes controlled remodelling continuously. In fact, dysregulated ECM remodelling is linked to pathological conditions and may aggravate disease progression. For instance, abnormal ECM deposition and stiffness are noticed in fibrosis and cancer,^44^ while excessive ECM degradation is associated with osteoarthritis.^45^ S100A4 protein can stimulate the production of ECM structural molecules and ECM remodelling proteases, in turn, plays an important regulatory role in the process of ECM remodelling.^34^ The main action of extracellular S100A4 during ECM remodelling is to stimulate the production of MMPs.^35^, ^43^, ^46^ MMPs as proteolytic enzymes play an essential role in ECM degradation process and greatly promote the inflammatory milieu.^47^ Evidence shows that the extracellular S100A4 can stimulate mouse endothelial cells and osteosarcoma cells to produce MMP‐13.^43^, ^48^ Extracellular S100A4 stimulates the increased production of MMPs (MMP‐1, −2, −3 and −9) and phenotypic transition of smooth muscle cells.^49^ Articular chondrocytes produce MMP‐13 in response to extracellular S100A4‐dependent activation,^46^ while human endothelial cells produce MMP‐9.^50^ Osteoarthritis synovial fibroblasts treated with S100A4 oligomer can induce the expression and release of MMP‐3 and other MMPs (MMP‐1, MMP‐9 and MMP‐13) from synovial fluid.^25^ Topical addition of recombinant S100A4 protein to mouse mammy epithelial cells can induce a significant increase in expression of MMP‐3, promoting mammary gland branching morphogenesis.^51^

In addition to the above functions, extracellular S100A4 also shows the ability to inhibit autophagy in lung cancer cells through activating β‐catenin signalling pathway in a RAGE‐dependent manner.^52^ Autophagy is the natural, regulated mechanism of the cell that removes unnecessary or dysfunctional components. It allows the orderly degradation and recycling of cellular components. Deregulation of autophagy is now accepted as a feature leading to the development of fibrosis.^53^, ^54^ Moreover, extracellular S100A4 is found to stimulate angiogenesis by interacting with annexin A2 on the surface of endothelial cells.^55^ Of course, there is another argument that extracellular S100A4 exerts its angiogenic ability through interaction with RAGE.^50^ Extracellular S100A4 stimulates endothelial cells to produce MMPs, indicating that ECM remodelling is the basis for S100A4 to stimulate endothelial cell movement and invasion.^43^, ^50^ The formation of new blood vessels (angiogenesis) is a process strictly related to progressive fibrogenesis, which leads to organ fibrosis.^56^ The growth of new blood vessels is also a required part of inflammatory processes.^56^

Besides, a recent study found that binding of extracellular S100A4 to embigin (a transmembrane glycoprotein belonging to the immunoglobulin superfamily) mediated prostate cancer progression by inhibition of adenosine monophosphate‐activated protein kinase (AMPK) activity, activation of nuclear factor kappa‐light‐chain‐enhancer of activated B cells (NF‐κB), MMP‐9 and mammalian target of rapamycin complex 1 (mTORC1) signalling, and inhibition of autophagy, which increased prostate cancer cell motility.^57^ In addition, in the context of fibrosis development, extracellular S100A4 could activate epidermal growth factor receptor (EGFR)/ErbB2 receptor signalling and improve the proliferation of mouse embryonic fibroblasts.^58^

### 3.3 Extracellular S100A4 as a damage‐associated molecular pattern (DAMP) protein

With the exception of embryos cell death caused by tissue damage always leads to inflammation. Inflammation is involved both in tissue regeneration and development of fibrosis.^59^, ^60^ In damaged tissues, inflammatory stimuli mainly come from the nucleus, cytosol, ECM or mitochondria of the necrotic cells and many factors such as high‐mobility group protein B1 (HMGB1), heat shock proteins, S100 protein (S100A8/S100A9) and heparan sulphate.^61^ These factors are known as ‘danger‐associated molecular patterns (DAMPs)’ and the corresponding receptors, called ‘pattern recognition receptors (PPRs)’ have been confirmed and included C‐type lectin receptors (CLRs), Retinoic acid inducible gene‐like receptors (RLRs), NOD‐like receptors (NLRs), RAGE and Toll‐like receptors (TLRs).^60^ DAMPs are usually stimulated to be released from the cell into the extracellular space. They bind to specific receptors on immune cells, thereby promoting the activation of innate immunity, cell differentiation and death, or the secretion of inflammatory mediators, further magnifying the inflammatory response.^62^

The S100A4 protein has been confirmed to be expressed in multiple cell types of lymphoid and myeloid lineages, including macrophages, neutrophils, mast cells and memory T cells, indicating a role of S100A4 in the regulation of immune cells.^32^, ^63^ As said before, S100A4 was found to be secreted by a subpopulation of inflammatory macrophages and consequently promotes the development of liver fibrosis and lung fibrosis.^14^, ^15^, ^20^ In vitro cultured human macrophages release S100A4 into extracellular space. Since increased level of S100A4 was observed in a tumour interstitial fluid of breast cancer, immune cells, at least of myeloid origin, are thought to be the source of extracellular S100A4 in the damaged tissues.^63^ It could be speculated that one of the ways S100A4 interferes with the body's immune response is its secretion from immune system cells in response to stress. Furthermore, extracellular S100A4 is a well‐known activator of pro‐inflammatory pathways and stimulator of cytokine production from various cells. Extracellular S100A4 exerts its effects on immune cells through affecting differentiation of cells of innate and adaptive immune systems and activating these cells to produce countless cytokines which most of them related to pro‐tumorigenic functions. Monocytes activated by extracellular S100A4 produced inflammatory cytokines which lead to aggravated disease activity of rheumatoid arthritis.^64^, ^65^ Likewise, extracellular S100A4 indirectly interfering with the differentiation of myeloid cells by activating breast cancer cells to elevate secretion of pro‐inflammatory cytokines that convert monocytes into the tumour‐associated macrophages.^66^ Moreover, in vitro studies cultured T cells in the presence of S100A4 alter the T cell polarization balance toward the Th2 phenotype,^67^ suggesting that extracellular S100A4 can affect T cell differentiation. Nevertheless, among the factors inducing fibrosis Th2 cytokines were the first to be confirmed to have strong profibrotic properties.^61^ Additionally, in recent years, we also paid attention to the role of S100A4 as a survival factor for myeloid‐derived suppressor cells (MDSCs).^68^ MDSCs represent a pathologic state of activation of monocytes and immature neutrophils. Extracellular S100A4 activated TLR‐4/ERK1/2 pathway to abrogate intrinsic caspase‐9‐dependent apoptosis induction in MDSCs, thus supporting their survival.^68^ A growing body of evidence shows clearly that the accumulation of MDSCs in tissues causes reduced collagen degradation and promotes organ fibrosis, including liver ^69^ and lung.^70^ Studies have confirmed that the interaction of recombinant or cell‐derived extracellular S100A4 with RAGE promotes ERK1/2 phosphorylation and activates the pro‐inflammatory NF‐κB axis.^40^, ^71^ Activation of NF‐κB axis directly and indirectly leads to the secretion of pro‐fibrogenic and pro‐proliferative factors, which result in fibrogenesis.^72^ In summary, extracellular S100A4‐mediated activation of cells from the innate and adaptive immune system is manifested by the production of cytokines. This network of cytokines consequently modulates the body's immune response.

According to the existing research on the biological characteristics of extracellular S100A4, it can be categorized as an effective DAMP protein, but its specific functions and cellular effects need to be further explored in the future. Extracellular S100A4 plays a substantial role in modulation of the immune system in many different ways also indicating that this protein could be a key player in a multitude of fibrotic diseases where the inflammatory pathway(s) play a large contributing role.

### 3.4 Specificity of extracellular S100‐receptor interactions

S100 proteins form heterodimers. These complexes display different affinities to target protein, depending on their oligomerization state.^73^ Multiple S100 proteins bind TLR4 ^65^ and RAGE.^73^ Structural analysis of ligand‐receptor interaction has suggested that RAGE recognizes three‐dimensional structures: one ‘V‐type’ domain and two ‘C‐type’ domains. The V‐type domain has been found to be responsible for ligand binding.^74^ RAGE has been shown to transform the extracellular effects of many S100 proteins, such as S100B, S100A1, S100A4 and S100A12.^75^

The interaction of S100B with RAGE has been demonstrated in many cell‐types. In RAGE overexpressing myocytes, S100B (100 nM) resulted in increases in VEGF mRNA, VEGF protein, VEGF secretion and activation of the transcription factor NF‐κB.^76^ In human SH‐SY5Y neuroblastoma cells, high concentration of S100B (5 μM) promoted cell survival through the PI3K/Akt/NF‐κB pathway in a RAGE‐dependent manner.^77^ Previous studies have shown that the interaction of dimeric S100B to RAGE to the isolated V‐domain and confirmed specific interaction between S100B and RAGE with sub‐micromolar affinity (K_D_≈0.5 μM).^78^ As for S100A1, binding to RAGE was observed in the micromolar range and strictly calcium dependent using surface plasmon resonance (SPR) technology.^79^ Recombinant S100A1 protein (100 nM) was shown to promote neurite outgrowth and to activate the transcription factor NF‐κB in a RAGE‐dependent manner.^80^ Mentioned S100A4, it interacts with RAGE in vitro as demonstrated also by SPR technology using either chimeric sRAGE‐Fc or biotinylated RAGE (K_D_ = 138 nM^81^;). Using the same technology but a GST‐RAGE fusion protein as a choice of binding partner, the results showed affinities in the same order of magnitude and strict calcium dependency for this interaction.^79^ The addition of recombinant S100A4 protein (300‐1500 ng/ml) resulted in endothelial dysfunction in a RAGE‐dependent manner.^26^ Similarly, recombinant S100A4 protein at 500 nM binds to RAGE to significantly promote human thyroid cancer cells migration.^41^ The inhibitory effect of S100A12 (10 μg/ml) on human foetal lung fibroblast migration is RAGE‐dependent, but not TLR4.^82^ SPR was used to measure the interaction of S100A12 with RAGE and the results revealed a submicromolar binding affinity between tetrameric S100A12 and the V‐domain.^79^

## 4 EXTRACELLULAR S100A4 IN DIAGNOSTICS

To date, several studies have proposed speculation as to whether extracellular S100A4 could be a prognostic biomarker for fibrotic diseases. Firstly, Yan et al clarified diagnostic accuracy of serum S100A4 level in patients with chronic hepatitis B (CHB).^83^ They found serum S100A4 level was higher in CHB patients with liver fibrosis. Using receiver operating characteristic (ROC) analyses, the area under the curves (AUC), sensitivity, specificity and accuracy of S100A4 were found to be 0.749, 62.7%, 75.9% and 0.70 for significant fibrosis (≥Stage 2), respectively. Secondly, increased serum S100A4 level was detected in IPF patients (27.3%).^84^ IPF patients with higher serum S100A4 levels had a significantly worse prognosis than those with low serum levels (2‐year cumulative survival rate: 41.7% vs. 77.0%, respectively). On multivariate analyses, baseline serum S100A4 levels (per 10 ng/ml increase) were independently associated with higher disease progression rate and higher mortality. S100A4 is a promising serum biomarker that may predict disease progression and mortality in IPF patients. Thirdly, extracellular S100A4 is strongly up‐regulated by immune cells and tissue‐resident cells at sites of inflammation, particularly in synovial fluid of patients with RA.^28^ Furthermore, increased S100A4 levels were present in the serum of patients with early RA (≤6 months duration), in whom persistently raised serum S100A4 was associated with a poor response to methotrexate. In patients with established RA (2‐44 years duration), increased serum levels of S100A4 indicated severe radiographic damage and non‐ response to infliximab therapy.^23^ These data indicated that high S100A4 levels were associated with a poor clinical response to infliximab and a high rate of anti‐infliximab antibodies. Another study also found that serum S100A4 levels were significantly higher in patients with early RA than in the healthy subjects and significantly decreased after 3 months of treatment.^85^ However, in female patients, high levels of S100A4 after 3 months of treatment predicted a worse outcome after 12 months of treatment (sensitivity of 56% and specificity of 94%). Persistently, high serum S100A4 levels predicted poor treatment outcome and S100A4 may thus represent a promising biomarker for assessing treatment response in patients with RA.

## 5 EXTRACELLULAR S100A4 AS A THERAPEUTIC TARGET

**Figure 2:**
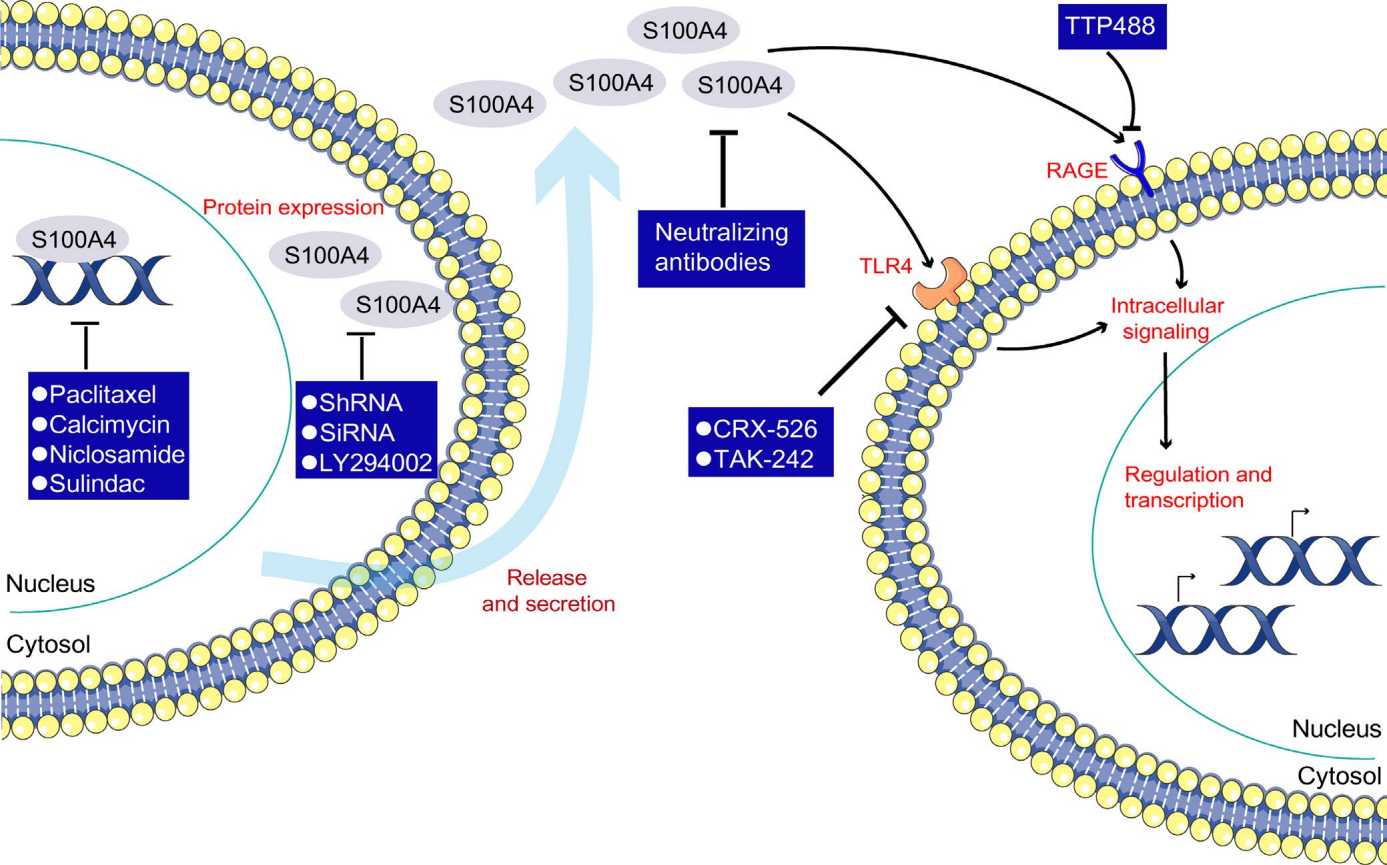
Targeting S100A4 protein function as potential therapies. S100A4 protein that has been released into the extracellular space exerts a vast array of activities, which presents a broad range of potential therapeutic strategies, including the inhibition of S100A4 protein expression, the prevention of S100A4 protein secretion and blocking S1004 protein‐receptor interactions. The expression and secretion of S100A4 can be effective reduced by the use of specific short hairpin RNA (shRNA) molecules and S100A4‐specific siRNA. Besides, LY294002 treatment can decrease S100A4 expression and secretion through inhibiting PI3K/Akt pathway. In addition, paclitaxel can reduce S100A4 expression by decreasing S100A4 nuclear import. Calcimycin, niclosamide and sulindac can mediate S100A4 expression through reducing S100A4 transcription. Pattern recognition receptors can also directly be targeted. TLR4 can be blocked by the CRX‐526, a synthetic lipid A mimetic molecule, and a small molecule inhibitor TAK‐242 (also called resatorvid). RAGE can be blocked by the small‐molecule inhibitor TTP488. In addition, S100A4 can be blocked by S100A4‐neutralizing antibodies. RAGE, receptor for advanced glycosylation end products; TLR4, Toll‐like receptor 4

Considerable progress has been made in the specific blockade of cytokines or their downstream signalling pathways for the treatment of fibrotic diseases; however, these therapies often have adverse effects; a substantial number of patients do not respond satisfactorily and long‐lasting remission is generally not achieved after medication is withdrawn. An urgent need still exists for innovative therapeutic approaches. Elevated extracellular S100A4 level contributes to the development and progression of many fibrotic diseases, implying that extracellular S100A4 could be a potential therapeutic target for treating fibrosis pathologies. In addition, extracellular S100A4 can function as a DAMP protein, thereby triggering pro‐inflammatory responses via binding to PRRs. Therefore, a wide range of treatment strategies need to be proposed, including inhibition of S100A4 protein expression and secretion, blocking S100A4 protein‐receptor interactions and reducing inflammatory response induced by S100A4 protein (Figure 2).

Several studies are currently available that confirm the effective blocking of extracellular S100A4 protein expression in vivo. The utilization of specific hairpin RNA to decrease the expression of S100A4 in CD11b‐positive macrophages from mice with liver fibrosis led to a reduction in the expression of α‐SMA and collagen deposition.^15^ However, owing to the high expression of S100A4 in myeloid cells, translation of such a strategy into a therapeutic application seems questionable. In another study, we used the neutralizing S100A4‐specific antibody clone 3B11 (10 µg/ml) to effectively inhibit the expression of extracellular S100A4 in mice with pulmonary fibrosis and the secretion from CD11b^+^F4/80^+^ macrophages.^20^ Additionally, another S100A4‐neutralizing antibody (6B12) was also found to inhibit the expression of extracellular S100A4, thereby altering T cell polarity balance.^67^ In the other study on extracellular S100A4 and pulmonary fibrosis, researchers used niclosamide (20 mg/kg) to inhibit the expression of extracellular S100A4 in mice with pulmonary fibrosis, while using S100A4‐specific siRNA to inhibit the secretion of S100A4 from M2 macrophages.^21^ Moreover, LY294002 (2‐(4‐Morpholinyl)‐8‐phenyl‐4H‐1‐benzopyran‐4‐one), a specific inhibitor of phosphatidylinositol 3‐hydroxy kinase (PI3K), could markedly decrease S100A4 expression in lung and S100A4 secretion in bronchoalveolar lavage fluid (BALF) in asthmatic mice.^86^ Thus, blocking the secretion of S100A4 protein via the alternative secretion pathway might be a promising approach for the treatment of fibrotic diseases.

Experimental data indicate that targeting PRRs might be a therapeutic option in fibrotic diseases. The TLR4 antagonist CRX‐526, a synthetic lipid A mimetic molecule, also known as an aminoalkyl‐glucosaminide‐phosphate (AGP), has therapeutic potential to attenuate renal injuries and slow the progression of advanced diabetic nephropathy in wild‐type and endothelial nitric oxide synthase (eNOS) knockout mice.^87^ Moreover, TAK‐242 (also called resatorvid, ethyl (6R)‐6‐[N‐(2‐chloro‐4‐fluorophenyl)sul‐famoyl]cyclohex‐1‐ene‐1‐carboxylate), a small molecule TLR4 inhibitor, could prevent, promote regression of bleomycin‐induced dermal and pulmonary fibrosis, and reduce the expression of several pro‐fibrotic mediators.^88^, ^89^ However, due to the parallel inhibition of antimicrobial recognition, blocking TLR4 may have a risk of significant adverse effects. Small molecule inhibitors such as TTP488 (3‐[4‐[2‐butyl‐1‐[4‐(4‐chlorophenoxy)phenyl]imidazol‐4‐yl]phenoxy]‐N,N‐diethylpropan‐1‐amine) have been developed that target the extracellular ligand‐binding site of RAGE.^90^ TTP488 inhibits the binding of multiple RAGE ligands, including advanced glycation end products, HMGB1, S100B and amyloid‐β.^90^ However, most pre‐clinical studies that block RAGE have focused on diabetic complications, cardiovascular disease and cancer; no published data have shown to effectively block RAGE in fibrotic diseases.^91^

Furthermore, small‐molecule inhibitors have also been developed that specifically block the interaction of S100A4 with target proteins. Simvastatin was reported to up‐regulate the expression of Annexin A10 protein but down‐regulate S100A4 expression.^92^ Low‐dose paclitaxel could reduce S100A4 nuclear import.^93^ Several compounds (such as calcimycin‐a calcium ionophore; niclosamide‐an antihelminth drug; and sulindac‐a non‐steroidal anti‐inflammatory drug) were observed to block the formation of the β‐catenin/T cell factor complex, which could directly mediate S100A4 transcription.^94^, ^95^, ^96^ Niclosamide, an FDA approved drug, is currently under evaluation for safety and efficacy in a phase II clinical trial for patients with metastatic colorectal cancer.^97^ Although these compounds do not focus on fibrosis, overlapping in the cell types producing S100A4 make them interesting for the fibrotic diseases. The class of small molecular S100A4 inhibitors with high affinity to S100A4 comprises the most likely candidates to be developed to pharmacologically control fibrosis in the near future.

## 6 CONCLUSION AND FUTURE EXPECTATIONS

The multifaceted roles of S100A4 in the development of cancer have been identified in plenty of studies.^8^, ^98^ It has been 10 years passed since the last published review on S100A4 in fibrosis y.^13^ In recent years, more evidence showed that S100A4 was involved in the development of tissue fibrosis, especially soluble S100A4 getting more attention from more scholars. Extracellular S100A4 is highly elevated at sites of inflammation in many fibrotic diseases. Once released in response to different stress and tissue damage, it functions as a DAMP protein, promoting sustained inflammation and leading to the destruction of organ structure. Owing to its local pattern of expression and release, S100A4 is identified as an excellent biomarker for monitoring disease activity in several fibrotic diseases. Insights into the mode of extracellular S100A4 function are uncovering novel molecular targets that drive innate and adaptive immune mechanisms of relevance to several fibrotic diseases. Overall, as a result of its local expression, mode of secretion and mechanisms of action, extracellular S100A4 is a promising target for future strategies to treat inflammatory fibrotic diseases.

Of course, we have to admit that there are still many issues to be resolved regarding extracellular S100A4. S100A4 can act degenerative or regenerative; it can be a cause or result of fibrotic disease progression. These questions are so far not answered comprehensively. The multifunctional character of S100A4, functioning intra‐ and extracellularly, may impede its suitability as a therapeutic target. S100A4 in‐ and outside of cells display mediate cell movement via various mechanisms, however, cardio‐ and neuroprotective effects may depend solely on extracellular S100A4. That being a guess, an up‐regulation in intracellular S100A4 protein may lead to more S100A4 release into extracellular, but only after injury or disease. In order to have a better understanding of these mechanisms, it is necessary for us to further understand how S100A4 is released and how S100A4 interacts with specific or promiscuous receptors. Since extracellular S100A4 acts as a DAMP protein, it is a worthwhile problem for us to discuss whether its release is associated with necrosis, pyroptosis or activation of inflammasome during fibrosis in the future work.

We think that future research could focus on the following major topics: (i) Verification of extracellular S100A4 as a marker in early fibrosis development and predicting treatment responses; (ii) Definition of the specific mechanisms underlying extracellular S100A4 up‐regulation in fibrosis; (iii) Definition of the functional overlaps and differences for extracellular S100A4 in tumorgenesis, neoangiogenesis and fibrotic diseases; (iv) Evaluation and validation of targeting extracellular S100A4 through S100A4 specific compounds for the therapy in pre‐clinical and clinical settings.

## CONFLICTS OF INTEREST

The authors confirm that there are no conflicts of interest.

## AUTHOR CONTRIBUTIONS

Q.Z conceived and designed the study. LZ LY and LS collected and analysed experiment data. LZ wrote and prepared the original draft.

## Data Availability

The data that support the findings of this study are available from the corresponding author upon reasonable request.

## References

[1] Feng Y‐L , Chen D‐Q , Vaziri ND , Guo Y , Zhao Y‐Y . Small molecule inhibitors of epithelial‐mesenchymal transition for the treatment of cancer and fibrosis. Med Res Rev. 2020;40:54‐78. 31131921 10.1002/med.21596

[2] Chen DQ , Feng YL , Cao G , Zhao YY . Natural products as a source for antifibrosis therapy. Trends Pharmacol Sci. 2018;39:937‐952. 30268571 10.1016/j.tips.2018.09.002

[3] Wynn TA , Ramalingam TR . Mechanisms of fibrosis: therapeutic translation for fibrotic disease. Nat Med. 2012;18:1028‐1040. 22772564 10.1038/nm.2807 PMC3405917

[4] Lv W , Booz GW , Wang Y , Fan F , Roman RJ . Inflammation and renal fibrosis: Recent developments on key signaling molecules as potential therapeutic targets. Eur J Pharmacol. 2018;820:65‐76. 29229532 10.1016/j.ejphar.2017.12.016 PMC6733417

[5] Li S , Hong M , Tan HY , Wang N , Feng N . Insights into the role and interdependence of oxidative stress and inflammation in liver diseases. Oxid Med Cell Longev. 2016;2016:4234061. 28070230 10.1155/2016/4234061 PMC5192343

[6] Nguyen‐Thanh T , Kim D , Lee S , Kim W , Park SK , Kang KP . Inhibition of histone deacetylase 1 ameliorates renal tubulointerstitial fibrosis via modulation of inflammation and extracellular matrix gene transcription in mice. Int J Mol Med. 2018;41:95‐106. 29115561 10.3892/ijmm.2017.3218 PMC5746318

[7] Liu Y . Cellular and molecular mechanisms of renal fibrosis. Nat Rev Nephrol. 2011;7(12):684‐696. 22009250 10.1038/nrneph.2011.149 PMC4520424

[8] Bresnick AR , Weber DJ , Zimmer DB . S100 proteins in cancer. Nat Rev Cancer. 2015;15:96‐109. 25614008 10.1038/nrc3893 PMC4369764

[9] Austermann J , Spiekermann C , Roth J . S100 proteins in rheumatic diseases. Nat Rev Rheumatol. 2018;14:528‐541. 30076385 10.1038/s41584-018-0058-9

[10] Fei F , Qu J , Li C , Wang X , Li Y , Zhang S . Role of metastasis‐induced protein S100A4 in human non‐tumor pathophysiologies. Cell Biosci. 2017;7:64. 29204268 10.1186/s13578-017-0191-1 PMC5702147

[11] Chow KH , Park HJ , George J , et al. S100A4 Is a biomarker and regulator of glioma stem cells that is critical for mesenchymal transition in glioblastoma. Cancer Res. 2017;77:5360‐5373. 28807938 10.1158/0008-5472.CAN-17-1294 PMC5626628

[12] Dahlmann M , Kobelt D , Walther W , Mudduluru G . Stein U. S100A4 in Cancer Metastasis: Wnt Signaling‐Driven Interventions for Metastasis Restriction. Cancers (Basel). 2016;8. 10.3390/cancers8060059 PMC4931624 27331819

[13] Schneider M , Hansen JL , Sheikh SP . S100A4: a common mediator of epithelial‐mesenchymal transition, fibrosis and regeneration in diseases? J Mol Med (Berl). 2008;86:507‐522. 18322670 10.1007/s00109-007-0301-3

[14] Osterreicher CH , Penz‐Osterreicher M , Grivennikov SI , et al. Fibroblast‐specific protein 1 identifies an inflammatory subpopulation of macrophages in the liver. Proc Natl Acad Sci U S A. 2011;108:308‐313. 21173249 10.1073/pnas.1017547108 PMC3017162

[15] Chen L , Li J , Zhang J , et al. S100A4 promotes liver fibrosis via activation of hepatic stellate cells. J Hepatol. 2015;62:156‐164. 25111176 10.1016/j.jhep.2014.07.035

[16] Dn OD , Sl A , Bb M . Influences of innate immunity, autophagy, and fibroblast activation in the pathogenesis of lung fibrosis. Am J Physiol Lung Cell Mol Physiol. 2016;311:L590‐601. 27474089 10.1152/ajplung.00221.2016 PMC5142210

[17] Barron L , Gharib SA , Duffield JS . Lung pericytes and resident fibroblasts: busy multitaskers. Am J Pathol. 2016;186:2519‐2531. 27555112 10.1016/j.ajpath.2016.07.004 PMC5222977

[18] Xia H , Gilbertsen A , Herrera J , et al. Calcium‐binding protein S100A4 confers mesenchymal progenitor cell fibrogenicity in idiopathic pulmonary fibrosis. J Clin Invest. 2017;127:2586‐2597. 28530639 10.1172/JCI90832 PMC5490771

[19] Lawson WE , Polosukhin VV , Zoia O , et al. Characterization of fibroblast‐specific protein 1 in pulmonary fibrosis. Am J Respir Crit Care Med. 2005;171:899‐907. 15618458 10.1164/rccm.200311-1535OC

[20] Li Y , Bao J , Bian Y , et al. S100A4(+) macrophages are necessary for pulmonary fibrosis by activating lung fibroblasts. Front Immunol. 2018;9:1776. 30127784 10.3389/fimmu.2018.01776 PMC6088238

[21] Zhang W , Ohno S , Steer B , et al. S100a4 Is secreted by alternatively activated alveolar macrophages and promotes activation of lung fibroblasts in pulmonary fibrosis. Front Immunol. 2018;9:1216. 29910813 10.3389/fimmu.2018.01216 PMC5992816

[22] Oslejskova L , Grigorian M , Gay S , Neidhart M , Senolt L . The metastasis associated protein S100A4: a potential novel link to inflammation and consequent aggressive behaviour of rheumatoid arthritis synovial fibroblasts. Ann Rheum Dis. 2008;67:1499‐1504. 18056757 10.1136/ard.2007.079905

[23] Erlandsson MC , Forslind K , Andersson SE , Lund A , Bokarewa MI . Metastasin S100A4 is increased in proportion to radiographic damage in patients with RA. Rheumatology (Oxford). 2012;51:932‐940. 22258387 10.1093/rheumatology/ker362

[24] Senolt L , Cerezo LA , Sumova B , et al. High levels of metastasis‐inducing S100A4 protein and treatment outcome in early rheumatoid arthritis: data from the PERAC cohort. Biomarkers. 2015;20:47‐51. 25489637 10.3109/1354750X.2014.989544

[25] Senolt L , Grigorian M , Lukanidin E , et al. S100A4 is expressed at site of invasion in rheumatoid arthritis synovium and modulates production of matrix metalloproteinases. Ann Rheum Dis. 2006;65:1645‐1648. 17105852 10.1136/ard.2005.047704 PMC1798462

[26] Herwig N , Belter B , Pietzsch J . Extracellular S100A4 affects endothelial cell integrity and stimulates transmigration of A375 melanoma cells. Biochem Biophys Res Commun. 2016;477:963‐969. 27387233 10.1016/j.bbrc.2016.07.009

[27] Rasanen K , Sriswasdi S , Valiga A , et al. Comparative secretome analysis of epithelial and mesenchymal subpopulations of head and neck squamous cell carcinoma identifies S100A4 as a potential therapeutic target. Mol Cell Proteomics. 2013;12:3778‐3792. 24037664 10.1074/mcp.M113.029587 PMC3861723

[28] Klingelhofer J , Senolt L , Baslund B , et al. Up‐regulation of metastasis‐promoting S100A4 (Mts‐1) in rheumatoid arthritis: putative involvement in the pathogenesis of rheumatoid arthritis. Arthritis Rheum. 2007;56:779‐789. 17328050 10.1002/art.22398

[29] Gong XJ , Song XY , Wei H , Wang J , Niu M . Serum S100A4 levels as a novel biomarker for detection of acute myocardial infarction. Eur Rev Med Pharmacol Sci. 2015;19:2221‐2225. 26166646

[30] Christensen MH , Fenne IS , Nordbo Y , et al. Novel inflammatory biomarkers in primary hyperparathyroidism. Eur J Endocrinol. 2015;173:9‐17. 25850829 10.1530/EJE-14-1038

[31] Kalluri R , Zeisberg M . Fibroblasts in cancer. Nat Rev Cancer. 2006;6:392‐401. 16572188 10.1038/nrc1877

[32] Weatherly K , Bettonville M , Torres D , Kohler A , Goriely S , Braun MY . Functional profile of S100A4‐deficient T cells. Immun Inflamm Dis. 2015;3:431‐444. 26734465 10.1002/iid3.85 PMC4693724

[33] Greene WA , Burke TA , Por ED , Kaini RR , Wang HC . Secretion profile of induced pluripotent stem cell‐derived retinal pigment epithelium during wound healing. Invest Ophthalmol Vis Sci. 2016;57:4428‐4441. 27750286 10.1167/iovs.16-19192

[34] Donato R , Cannon BR , Sorci G , et al. Functions of S100 proteins. Curr Mol Med. 2013;13:24‐57. 22834835 PMC3707951

[35] Schmidt‐Hansen B , Klingelhofer J , Grum‐Schwensen B , et al. Functional significance of metastasis‐inducing S100A4(Mts1) in tumor‐stroma interplay. J Biol Chem. 2004;279:24498‐24504. 15047714 10.1074/jbc.M400441200

[36] Yammani RR , Long D , Loeser RF . Interleukin‐7 stimulates secretion of S100A4 by activating the JAK/STAT signaling pathway in human articular chondrocytes. Arthritis Rheum. 2009;60:792‐800. 19248116 10.1002/art.24295 PMC2676111

[37] Forst B , Hansen MT , Klingelhofer J , et al. Metastasis‐inducing S100A4 and RANTES cooperate in promoting tumor progression in mice. PLoS ONE. 2010;5:e10374. 20442771 10.1371/journal.pone.0010374 PMC2860983

[38] Dukhanina EA , Portseva TN , Pankratova EV , et al. Oct‐1 modifies S100A4 exchange between intra‐ and extracellular compartments in Namalwa cells and increases their sensitivity to glucocorticoids. Cell Cycle. 2016;15:1471‐1478. 27096393 10.1080/15384101.2016.1175260 PMC4934052

[39] Smith BN , Bhowmick NA . Role of EMT in metastasis and therapy resistance. J Clin Med. 2016;5(2):17. 10.3390/jcm5020017 PMC4773773 26828526

[40] Dahlmann M , Okhrimenko A , Marcinkowski P , et al. RAGE mediates S100A4‐induced cell motility via MAPK/ERK and hypoxia signaling and is a prognostic biomarker for human colorectal cancer metastasis. Oncotarget. 2014;5:3220‐3233. 24952599 10.18632/oncotarget.1908 PMC4102805

[41] Medapati MR , Dahlmann M , Ghavami S , et al. RAGE mediates the pro‐migratory response of extracellular S100A4 in human thyroid cancer cells. Thyroid. 2015;25:514‐527. 25744544 10.1089/thy.2014.0257

[42] Spiekerkoetter E , Guignabert C , de Jesus PV , et al. S100A4 and bone morphogenetic protein‐2 codependently induce vascular smooth muscle cell migration via phospho‐extracellular signal‐regulated kinase and chloride intracellular channel 4. Circ Res. 2009; 105: 639‐647. 13 p following 47. 19713532 10.1161/CIRCRESAHA.109.205120 PMC2818124

[43] Schmidt‐Hansen B , Ornas D , Grigorian M , et al. Extracellular S100A4(mts1) stimulates invasive growth of mouse endothelial cells and modulates MMP‐13 matrix metalloproteinase activity. Oncogene. 2004;23:5487‐5495. 15122322 10.1038/sj.onc.1207720

[44] Frantz C , Stewart KM , Weaver VM . The extracellular matrix at a glance. J Cell Sci. 2010;123:4195‐4200. 21123617 10.1242/jcs.023820 PMC2995612

[45] Zhen G , Cao X . Targeting TGFbeta signaling in subchondral bone and articular cartilage homeostasis. Trends Pharmacol Sci. 2014;35:227‐236. 24745631 10.1016/j.tips.2014.03.005 PMC4058854

[46] Yammani RR , Carlson CS , Bresnick AR , Loeser RF . Increase in production of matrix metalloproteinase 13 by human articular chondrocytes due to stimulation with S100A4: Role of the receptor for advanced glycation end products. Arthritis Rheum. 2006;54:2901‐2911. 16948116 10.1002/art.22042

[47] Nissinen L , Kahari VM . Matrix metalloproteinases in inflammation. Biochim Biophys Acta. 2014;1840:2571‐2580. 24631662 10.1016/j.bbagen.2014.03.007

[48] Berge G , Pettersen S , Grotterod I , Bettum IJ , Boye K , Maelandsmo GM . Osteopontin–an important downstream effector of S100A4‐mediated invasion and metastasis. Int J Cancer. 2011;129:780‐790. 20957651 10.1002/ijc.25735

[49] Chaabane C , Heizmann CW , Bochaton‐Piallat ML . Extracellular S100A4 induces smooth muscle cell phenotypic transition mediated by RAGE. Biochim Biophys Acta. 2015;1853:2144‐2157. 25110349 10.1016/j.bbamcr.2014.07.022

[50] Hernandez JL , Padilla L , Dakhel S , et al. Therapeutic targeting of tumor growth and angiogenesis with a novel anti‐S100A4 monoclonal antibody. PLoS ONE. 2013;8:e72480. 24023743 10.1371/journal.pone.0072480 PMC3762817

[51] Andersen K , Mori H , Fata J , et al. The metastasis‐promoting protein S100A4 regulates mammary branching morphogenesis. Dev Biol. 2011;352:181‐190. 21195708 10.1016/j.ydbio.2010.12.033 PMC3643517

[52] Hou S , Tian T , Qi D , et al. S100A4 promotes lung tumor development through β‐catenin pathway‐mediated autophagy inhibition. Cell Death Dis. 2018;9:277. 29449540 10.1038/s41419-018-0319-1 PMC5833421

[53] Song Y , Zhao Y , Wang F , Tao L , Xiao J , Yang C Autophagy in hepatic fibrosis. Biomed Res Int. 2014; 2014:436242. 24779010 10.1155/2014/436242 PMC3980865

[54] Zhao XC , Livingston MJ , Liang XL , Dong Z . Cell apoptosis and autophagy in renal fibrosis. Adv Exp Med Biol. 2019;1165:557‐584. 31399985 10.1007/978-981-13-8871-2_28

[55] Semov A , Moreno MJ , Onichtchenko A , et al. Metastasis‐associated protein S100A4 induces angiogenesis through interaction with Annexin II and accelerated plasmin formation. J Biol Chem. 2005;280:20833‐20841. 15788416 10.1074/jbc.M412653200

[56] Kreuger J , Phillipson M . Targeting vascular and leukocyte communication in angiogenesis, inflammation and fibrosis. Nat Rev Drug Discov. 2016;15:125‐142. 26612664 10.1038/nrd.2015.2

[57] Ruma I , Kinoshita R , Tomonobu N , et al. Embigin promotes prostate cancer progression by S100A4‐dependent and‐independent mechanisms. Cancers (Basel). 2018;10(7):239. 10.3390/cancers10070239 PMC6071117 30041429

[58] Klingelhofer J , Moller HD , Sumer EU , et al. Epidermal growth factor receptor ligands as new extracellular targets for the metastasis‐promoting S100A4 protein. Febs J. 2009;276:5936‐5948. 19740107 10.1111/j.1742-4658.2009.07274.x

[59] Eming SA , Wynn TA , Martin P . Inflammation and metabolism in tissue repair and regeneration. Science. 2017;356:1026‐1030. 28596335 10.1126/science.aam7928

[60] Roh JS , Sohn DH . Damage‐associated molecular patterns in inflammatory diseases. Immune Netw. 2018;18:e27. 30181915 10.4110/in.2018.18.e27 PMC6117512

[61] Mack M . Inflammation and fibrosis. Matrix Biol. 2018;68–69:106‐121. 10.1016/j.matbio.2017.11.010 29196207

[62] Boteanu RM , Suica VI , Uyy E , et al. Alarmins in chronic noncommunicable diseases: Atherosclerosis, diabetes and cancer. J Proteomics. 2017;153:21‐29. 27840210 10.1016/j.jprot.2016.11.006

[63] Cabezon T , Celis JE , Skibshoj I , et al. Expression of S100A4 by a variety of cell types present in the tumor microenvironment of human breast cancer. Int J Cancer. 2007;121:1433‐1444. 17565747 10.1002/ijc.22850

[64] Oslejskova L , Grigorian M , Hulejova H , et al. Metastasis‐inducing S100A4 protein is associated with the disease activity of rheumatoid arthritis. Rheumatology (Oxford). 2009;48:1590‐1594. 19828600 10.1093/rheumatology/kep316

[65] Cerezo LA , Remakova M , Tomcik M , et al. The metastasis‐associated protein S100A4 promotes the inflammatory response of mononuclear cells via the TLR4 signalling pathway in rheumatoid arthritis. Rheumatology (Oxford). 2014;53:1520‐1526. 24643522 10.1093/rheumatology/keu031

[66] Prasmickaite L , Tenstad EM , Pettersen S , et al. Basal‐like breast cancer engages tumor‐supportive macrophages via secreted factors induced by extracellular S100A4. Mol Oncol. 2018;12:1540‐1558. 29741811 10.1002/1878-0261.12319 PMC6120223

[67] Grum‐Schwensen B , Klingelhofer J , Beck M , et al. S100A4‐neutralizing antibody suppresses spontaneous tumor progression, pre‐metastatic niche formation and alters T‐cell polarization balance. BMC Cancer. 2015;15:44. 25884510 10.1186/s12885-015-1034-2 PMC4335362

[68] Li Q , Dai C , Xue R , et al. S100A4 protects myeloid‐derived suppressor cells from intrinsic apoptosis via TLR4‐ERK1/2 signaling. Front Immunol. 2018;9:388. 29556233 10.3389/fimmu.2018.00388 PMC5845385

[69] Weston CJ , Zimmermann HW , Adams DH . The role of myeloid‐derived cells in the progression of liver disease. Front Immunol. 2019;10:893. 31068952 10.3389/fimmu.2019.00893 PMC6491757

[70] Lebrun A , Lo Re S , Chantry M ,, et al. CCR2 monocytic myeloid‐derived suppressor cells (M‐MDSCs) inhibit collagen degradation and promote lung fibrosis by producing transforming growth factor‐β1. J Pathol. 2017;243:320‐330. 28799208 10.1002/path.4956

[71] Haase‐Kohn C , Wolf S , Herwig N , Mosch B , Pietzsch J . Metastatic potential of B16–F10 melanoma cells is enhanced by extracellular S100A4 derived from RAW264.7 macrophages. Biochem Biophys Res Commun. 2014;446:143‐148. 24613382 10.1016/j.bbrc.2014.02.126

[72] Elsharkawy AM , Mann DA . Nuclear factor‐kappaB and the hepatic inflammation‐fibrosis‐cancer axis. Hepatology. 2007;46:590‐597. 17661407 10.1002/hep.21802

[73] Palanissami G , Paul SFD . RAGE and its ligands: molecular interplay between glycation, inflammation, and hallmarks of cancer—a review. Horm Cancer. 2018;9:295‐325. 29987748 10.1007/s12672-018-0342-9 PMC10355895

[74] Sorci G , Riuzzi F , Giambanco I , Donato R . RAGE in tissue homeostasis, repair and regeneration. Biochim Biophys Acta. 2013;1833:101‐109. 23103427 10.1016/j.bbamcr.2012.10.021

[75] Ji YF , Huang H , Jiang F , Ni RZ , Xiao MB . S100 family signaling network and related proteins in pancreatic cancer (Review). Int J Mol Med. 2014;33:769‐776. 24481067 10.3892/ijmm.2014.1633

[76] Tsoporis JN , Izhar S , Proteau G , Slaughter G , Parker TG . S100B‐RAGE dependent VEGF secretion by cardiac myocytes induces myofibroblast proliferation. J Mol Cell Cardiol. 2012;52:464‐473. 21889514 10.1016/j.yjmcc.2011.08.015

[77] Leclerc E , Fritz G , Weibel M , Heizmann CW , Galichet A . S100B and S100A6 differentially modulate cell survival by interacting with distinct RAGE (receptor for advanced glycation end products) immunoglobulin domains. J Biol Chem. 2007;282:31317‐31331. 17726019 10.1074/jbc.M703951200

[78] Dattilo BM , Fritz G , Leclerc E , Kooi CW , Heizmann CW , Chazin WJ . The extracellular region of the receptor for advanced glycation end products is composed of two independent structural units. Biochemistry. 2007;46:6957‐6970. 17508727 10.1021/bi7003735 PMC2527459

[79] Leclerc E , Fritz G , Vetter SW , Heizmann CW . Binding of S100 proteins to RAGE: an update. Biochim Biophys Acta. 2009;1793:993‐1007. 19121341 10.1016/j.bbamcr.2008.11.016

[80] Huttunen HJ , Kuja‐Panula J , Sorci G , Agneletti AL , Donato R , Rauvala H . Coregulation of neurite outgrowth and cell survival by amphoterin and S100 proteins through receptor for advanced glycation end products (RAGE) activation. J Biol Chem. 2000;275:40096‐40105. 11007787 10.1074/jbc.M006993200

[81] Kiryushko D , Novitskaya V , Soroka V , et al. Molecular mechanisms of Ca(2+) signaling in neurons induced by the S100A4 protein. Mol Cell Biol. 2006;26:3625‐3638. 16612001 10.1128/MCB.26.9.3625-3638.2006 PMC1447425

[82] Tanaka N , Ikari J , Anazawa R , et al. S100A12 inhibits fibroblast migration via the receptor for advanced glycation end products and p38 MAPK signaling. Vitro Cell Dev Biol Anim. 2019;55:656‐664. 10.1007/s11626-019-00384-x 31297698

[83] Yan LB , Zhang QB , Zhu X , He M , Tang H . Serum S100 calcium binding protein A4 improves the diagnostic accuracy of transient elastography for assessing liver fibrosis in hepatitis B. Clin Res Hepatol Gastroenterol. 2018;42:64‐71. 28688902 10.1016/j.clinre.2017.05.013

[84] Akiyama N , Hozumi H , Isayama T , et al. Clinical significance of serum S100 calcium‐binding protein A4 in idiopathic pulmonary fibrosis. Respirology. 2019 10.1111/resp.13707 31597225

[85] Šenolt L , Cerezo LA , Šumová B , et al.. High levels of metastasis‐inducing S100A4 protein and treatment outcome in early rheumatoid arthritis: data from the PERAC cohort. Biomarkers. 2015;20(1):47‐51. 25489637 10.3109/1354750X.2014.989544

[86] Huang X , Qu D , Liang Y , Huang Q , Li M , Hou C . Elevated S100A4 in asthmatics and an allergen‐induced mouse asthma model. J Cell Biochem. 2019;120(6):9667‐9676. 30569582 10.1002/jcb.28245

[87] Fort MM , Mozaffarian A , Stöver AG , et al. A synthetic TLR4 antagonist has anti‐inflammatory effects in two murine models of inflammatory bowel disease. J Immunol. 1950;2005(174):6416‐6423. 10.4049/jimmunol.174.10.6416 15879143

[88] Matsunaga N , Tsuchimori N , Matsumoto T , Ii M . TAK‐242 (Resatorvid), a small‐molecule inhibitor of toll‐like receptor (TLR) 4 signaling, binds selectively to TLR4 and interferes with interactions between TLR4 and its adaptor molecules. Mol Pharmacol. 2011;79(1):34‐41. 20881006 10.1124/mol.110.068064

[89] Bhattacharyya S , Wang W , Tamaki Z , et al. Pharmacological inhibition of toll‐like receptor‐4 signaling by TAK242 prevents and induces regression of experimental organ fibrosis. Front Immunol. 2018;9:2434. 30405628 10.3389/fimmu.2018.02434 PMC6207051

[90] Sabbagh MN , Agro A , Bell J , Aisen PS , Schweizer E , Galasko D . PF‐04494700, an oral inhibitor of receptor for advanced glycation end products (RAGE), in alzheimer disease. Alzheimer Dis Assoc Disord. 2011;25(3):206‐212. 21192237 10.1097/WAD.0b013e318204b550 PMC3346183

[91] Bi H , Me L . Targeting RAGE Signaling in Inflammatory Disease. Annual Rev Med. 2018;69:349‐364. 29106804 10.1146/annurev-med-041316-085215

[92] Miyazawa Y , Sekine Y , Kato H , Furuya Y , Koike H , Suzuki K . Simvastatin up‐regulates annexin A10 that can inhibit the proliferation, migration, and invasion in androgen‐independent human prostate cancer cells. Prostate. 2017;77(4):337‐349. 27862098 10.1002/pros.23273

[93] Cadamuro M , Spagnuolo G , Sambado L , et al. Low‐dose paclitaxel reduces S100A4 nuclear import to inhibit invasion and hematogenous metastasis of cholangiocarcinoma. Cancer Res. 2016;76(16):4775‐4784. 27328733 10.1158/0008-5472.CAN-16-0188 PMC4987167

[94] Dahlmann M , Kobelt D , Walther W , Mudduluru G , Stein U . S100A4 in cancer metastasis: wnt signaling‐driven interventions for metastasis restriction. Cancers. 2016;8(6):59. 10.3390/cancers8060059 PMC4931624 27331819

[95] Sack U , Walther W , Scudiero D , et al. S100A4‐induced cell motility and metastasis is restricted by the Wnt/β‐catenin pathway inhibitor calcimycin in colon cancer cells. Mol Biol Cell. 2011;22(18):3344‐3354. 21795396 10.1091/mbc.E10-09-0739 PMC3172260

[96] Stein U , Arlt F , Smith J , et al. Intervening in β‐catenin signaling by sulindac inhibits s100a4‐dependent colon cancer metastasis. Neoplasia. 2011;13(2):131‐IN8. 21403839 10.1593/neo.101172 PMC3033592

[97] Burock S , Daum S , Keilholz U , Neumann K , Walther W , Stein U . Phase II trial to investigate the safety and efficacy of orally applied niclosamide in patients with metachronous or sychronous metastases of a colorectal cancer progressing after therapy: the NIKOLO trial. BMC Cancer. 2018; 18: 297. 29544454 10.1186/s12885-018-4197-9 PMC5856000

[98] Fei F , Qu J , Zhang M , Li Y , Zhang S . S100A4 in cancer progression and metastasis: a systematic review. Oncotarget. 2017;8:73219‐39. 29069865 10.18632/oncotarget.18016 PMC5641208

